# Effects of Blueberry Consumption on Fecal Microbiome Composition and Circulating Metabolites, Lipids, and Lipoproteins in a Randomized Controlled Trial of Older Adults with Overweight or Obesity: The BEACTIVE Trial

**DOI:** 10.3390/nu17071200

**Published:** 2025-03-29

**Authors:** Kathryn N. Porter Starr, Margery A. Connelly, Jessica Wallis, Rebecca North, Qimin Zhang, Kuncheng Song, Jessica M. González-Delgado, Hayden N. Brochu, Crystal R. Icenhour, Lakshmanan K. Iyer, Marshall G. Miller, Kim M. Huffman, William E. Kraus, Connie W. Bales

**Affiliations:** 1 Center for the Study of Aging and Human Development, Duke University School of Medicine, Durham, NC 27710, USA; jessica.wallis@duke.edu (J.W.); rebecca.thiem@duke.edu (R.N.); marshall.miller@duke.edu (M.G.M.); 2 Department of Medicine, Duke University School of Medicine, Durham, NC 27710, USA; kim.huffman@duke.edu (K.M.H.); william.kraus@duke.edu (W.E.K.); connie.bales@duke.edu (C.W.B.); 3 Geriatric Research, Education, and Clinical Center, Durham VA Medical Center, Durham, NC 27710, USA; 4 Labcorp, Morrisville, NC 27560, USA; connem5@labcorp.com (M.A.C.); gonzj59@labcorp.com (J.M.G.-D.); 5 Labcorp, Westborough, MA 01581, USA; zhangq3@labcorp.com (Q.Z.); songk2@labcorp.com (K.S.); brochuh@labcorp.com (H.N.B.); iyerl@labcorp.com (L.K.I.); 6 Labcorp, Burlington, NC 27710, USA; icenhoc@labcorp.com; 7 Department of Medicine, Duke Molecular Physiology Institute, Duke University School of Medicine, Durham, NC 27710, USA

**Keywords:** blueberry intake, gut microbiome, fecal microbiome, polyphenols, lipoproteins, cardiometabolic risk, nuclear magnetic resonance

## Abstract

**Background/Objectives:** Generous consumption of phytonutrient-rich foods, including blueberries, provides benefits to multiple physiologic and metabolic systems. This study explored the potential that regular, generous blueberry intake could favorably modulate fecal microbiome composition in sedentary older (>60 years) men and women with overweight or obesity (BMI ≥ 25 to 32 kg/m^2^). **Methods:** Participants (n = 55) were randomized to daily consumption of either lyophilized blueberry powder (equivalent to 1.5 cups of blueberries) or an indistinguishable placebo powder; both groups participated in weekly supervised exercise classes. Fecal samples were collected at 0 and 12 weeks and frozen. Following this, 16S rRNA gene sequencing was used to profile each participant’s fecal microbiome. Blood biomarkers of cardiometabolic health were measured via nuclear magnetic resonance spectroscopy (NMR) pre- and post-treatment. **Results:** Comparing the baseline and endpoint results for the blueberry (n = 15) and placebo (n = 19) groups, there were no significant overall compositional differences or differences in the level of diversity in the fecal microbiome. However, in subjects whose diet included blueberry powder, there was a significant enrichment (*p* = 0.049) in the relative abundance of *Coriobacteriales incertae sedis*, a taxonomic group of bacteria that facilitates the metabolism of dietary polyphenols. The placebo group exhibited significant reductions in total cholesterol, LDL-C, non-HDL-C, total LDL-P, large LDL-P, and ApoB, while the blueberry group exhibited significant reductions in total HDL-P and ApoA-I after 12 weeks compared to baseline. **Conclusions:** Generous blueberry consumption may upregulate the ability of the older human gut to utilize dietary polyphenols by altering the fecal microbiome. Longer, larger-scale studies with blueberries or blueberry powder are needed to observe improvements in cardiometabolic risk factors in older adults with overweight or obesity.

## 1. Introduction

Due to their bioactive constituents, increased consumption of blueberries, as well as other phytonutrient-rich foods, represents an excellent lifestyle enhancement for promoting health and wellness [1,2,3,4,5]. Of the most important classes of blueberry-derived bioactive substances, polyphenols, which include anthocyanins, are a subgroup of flavonoids responsible for the distinctive blueberry color. Dietary polyphenols, and blueberry anthocyanins in particular, act on multiple targets in the vascular system, with benefits including reduction of oxidative stress and inflammation, activation of endothelial nitric oxide synthase signaling, amelioration of dyslipidemia, and reductions in cardiometabolic risk factors [6,7,8,9,10].

The fecal microbiome plays an important role in maintaining host health and may influence the pathogenesis of various metabolic and inflammatory diseases, such as obesity, diabetes, and inflammatory bowel disease [2,3,4,5]. There is growing evidence in rodent models that increased dietary polyphenols, including those found in blueberries, directly modulates fecal microbiome composition, which may lead to improved health [11,12,13,14,15,16,17,18]. For example, changes in the fecal microbiome in rats on a high-fat diet with blueberry supplementation lead to an increase in polyphenol metabolism and improvements in glycemic control [18,19]. Dietary supplementation with blueberry powder in mice increases fecal *Coriobacteriaceae* and reduces circulating trimethylamine-N-oxide (TMAO), a marker of cardiovascular and mortality risk [20,21,22,23]. In diabetic mice or mice on an obesogenic diet, alterations in fecal microbiome content are associated with improvements in fatty acid and lipid metabolism, glucose tolerance, insulin sensitivity, and decreases in vascular inflammation with or without weight loss [11,13,14,15,17]. Therefore, increased dietary polyphenols may change the fecal microbiome in ways that promote cardiometabolic health.

Blueberry-enriched dietary studies in humans result in similar findings [24,25,26,27]. Six-week consumption of a diet including a blueberry powder drink increases *Bifidobacteria* bacteria in fecal samples [24]. In older, but not in younger, healthy women, whole blueberries and their isolated polyphenol-rich fractions increase the diversity in bacterial species within the gut [25]. Short-term blueberry supplementation leads to an improvement in glucose control and insulin levels in sedentary subjects [26]. Consumption of a phenol-rich apple/berry drink containing blueberry juice improves the lipid profile in women with overweight or obesity [27]. Together, these studies suggest that diets rich in blueberries or blueberry constituents modulate the fecal microbiome, leading to metabolic improvements in humans.

Lifestyle interventions that are practical, non-invasive, and unlikely to interact with common comorbidities and their treatments can be especially beneficial for older adults, who often face a variety of health challenges. The goal of this pilot study was to assess the impact of consumption of a diet enhanced with blueberry powder relative to a placebo control on the fecal microbiome and circulating lipids, lipoproteins, and other metabolites indicative of cardiovascular risk in older adults with overweight or obesity. This study links microbiome research to cardiovascular health by exploring the impact of gut-derived metabolites on lipid metabolism and biomarkers associated with cardiovascular disease (CVD). It advances our understanding of these mechanisms, paving the way for targeted interventions to improve gut health and lower cardiovascular risks.

## 2. Materials and Methods

### 2.1. BEACTIVE Study Design

Blueberry Enhances Activity and Cognition Through Increased Vascular Efficiency (BEACTIVE) was a 12-week randomized, double-blind, placebo-controlled trial with two arms: a blueberry consumption plus exercise intervention was compared to a placebo plus exercise intervention (NCT04049162). Sedentary (<30 min of moderate physical activity/week) older (>60 years) women and men with a body mass index (BMI) from ≥25 to 32 kg/m^2^ (overweight and moderate obesity) and a normal heart rhythm and blood pressure <140/90 mmHg were eligible for study enrollment. Subjects with chronic conditions were included; those taking medications other than statins for conditions associated with metabolic syndrome (hypertension, diabetes, dyslipidemia) or unwilling to restrict consumption of anthocyanin-rich foods were excluded. Additional exclusions included antibiotic use in the last 3 months, colonoscopy in the last 2 months, and history of frequent urinary tract or *Clostridium difficile* infections. This study was conducted according to the guidelines of the Declaration of Helsinki and approved by the Duke University Health System Institutional Review Board (protocol number: Pro00101714). Informed consent was obtained from all study participants.

**Figure 1:**
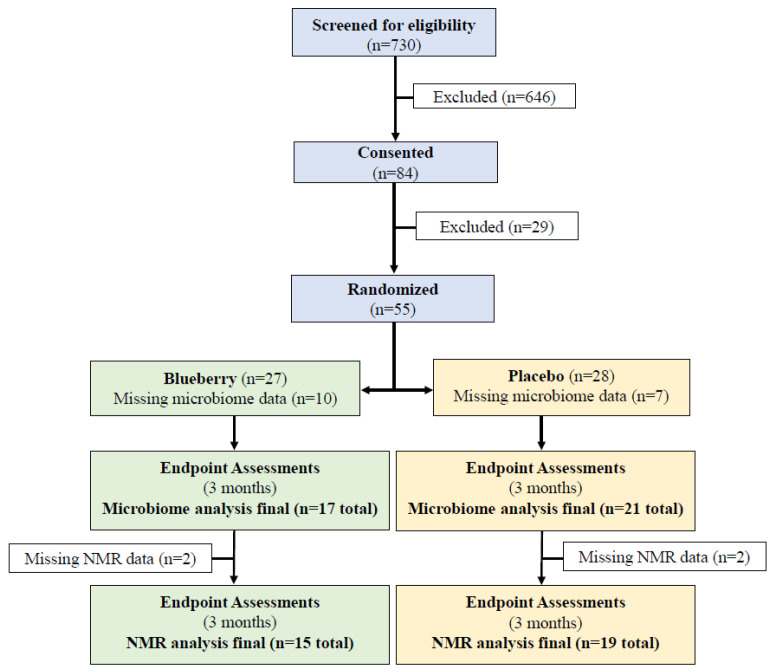
Consort diagram for the BEACTIVE study.

All subjects were recruited from the local community. Subjects (n = 730) were screened for eligibility (see consort diagram; Figure 1). Of these, 675 subjects were excluded, either for ineligibility criteria or study dropout (Figure 1). Participants (n = 55) were randomized to either lyophilized blueberry powder (18 g, equivalent to 3/4 cup of blueberries) rehydrated and consumed as a beverage twice daily with meals (n = 27) or an indistinguishable placebo powder taken in the same manner (placebo control, n = 28). A total of 48 subjects completed the study (n = 23 blueberry and n = 25 placebo) (Figure 1). The 12-week study duration was based upon the timing of vascular responses seen in other blueberry trials. The blueberry dose of 36 g per day (equivalent to 1.5 cups of blueberries and providing 1116 mg of phenolics and 382 mg of anthocyanins) in a split dose consumed with meals was based on (1) a 33% increase in dose over that previously used in a longer (6-month) trial; (2) delivery of the most effective dose of blueberry bioactives, such as anthocyanins [28]; and (3) reduced likelihood of any gastrointestinal symptoms.

### 2.2. Clinical Assessments

At baseline, participant diets were evaluated for nutrient content and berry consumption using a 3-day diet record and a food frequency questionnaire administered via an online program (DHQ-III: https://epi.grants.cancer.gov/dhq3/; URL accessed on 6 January 2020); a berry-specific rider was added to the DHQ-III. To maximize the ability to distinguish treatment impact, eligible participants underwent a 2-week washout phase prior to randomization. Subjects were asked to abstain from berry fruits and other anthocyanin-rich foods (berries/grapes/cherries, berry juice, wine, vinegar, dried berries/raisins, or products containing them) during the washout period and for the duration of the trial.

Both treatment groups were asked to increase physical activity by participating in a weekly supervised exercise class and to increase baseline daily step count gradually over the course of the intervention period. At baseline, participants were asked to wear a Garmin device for 7 days, and an average baseline step count was determined and rounded up to the nearest 250 steps. Participants were asked to increase their step count by at least 750 steps each month. Vascular function, 24-r ambulatory blood pressure, cognitive performance, and related secondary measures were assessed at 0 and 12 weeks. Berry and nutrient intake were assessed every 4 weeks, and physical activity as step count per day was continuously monitored using a mobile device (Garmin). Blood and stool samples were collected at 0 and 12 weeks and stored at <−70 °C for later analysis of fecal microbiomes via 16S rRNA gene sequencing (stool), lipoprotein profiling (blood), and metabolite concentrations (blood) via nuclear magnetic resonance (NMR) spectroscopy.

### 2.3. Fecal Microbiome Characterization

Of the study participants, 17 in the blueberry powder group and 21 in the placebo group had fecal samples collected before (baseline) and after (endpoint) treatment, resulting in 76 samples from 38 participants for inclusion in fecal microbiome characterization (Figure 1). To characterize fecal microbiomes, fecal samples were thawed, fecal material was collected onto a flocked swab, and the sample was placed in a DNA/RNA Shield (Zymo Research, Irvine, CA, USA). Samples underwent DNA extraction using the ZymoBIOMICS 96 MagBead DNA isolation kit, as directed by the manufacturer (Zymo Research, Irvine, CA, USA). The 16S rRNA gene V3-V4 hypervariable region was amplified using 341f (5′-CCTACGGGNGGCWGCAG-3′) and 805r (5′-GACTACHVGGGTATCTAATCC-3′) primers. Library preparation was performed using the KAPA HyperPrep and KAPA Library amplification kits, as directed by the manufacturer (Roche Diagnostics Corporation, Indianapolis, IN, USA); the resulting libraries were sequenced on an Illumina MiSeq platform (Illumina Inc., San Diego, CA, USA) using 300 base pair (bp) paired-end reads. Then, 16S rRNA gene V3-V4 sequencing reads were processed using R v4.2.0 [29] by first reorienting reads to the same strand and then trimming using the DADA2 [30] v1.22.0 filterAndTrim function with default parameters and trimRight=c(68, 52) (optimally chosen using in-house R function). Quality-filtered trimmed reads were then processed through the standard DADA2 process, denoising and merging reads to generate amplicon sequence variants (ASVs). ASVs were then filtered by removing singletons and those with lengths shorter than 350 bp. The resulting ASVs were taxonomically classified using the SILVA v138 database [31] and DADA2-implemented RDP classifier [32] with a bootstrap confidence threshold of 80. ASVs without at least a phylum rank classification were removed from analysis. A final taxonomically aggregated count matrix was then generated by aggregating ASVs at the lowest assigned taxonomic rank. Sequencing quality and number of reads for each sample was assessed.

### 2.4. NMR Quantification of Lipoproteins, Lipids, and Small Molecule Metabolites Associated with Cardiovascular Risk

Frozen EDTA-plasma samples were thawed and measured using a Vantera^®^ Clinical Analyzer (Labcorp, Morrisville, NC, USA), a fully automated, high-throughput, 400 MHz proton (1H) NMR spectroscopy platform. NMR spectral data acquisition on the Vantera^®^ Clinical Analyzer, spectra data processing, and quantification of TMAO, betaine, and choline have been reported in greater detail elsewhere [33,34,35]. Lipoprotein profile results were calculated using the LP4 algorithm, as previously described. Details of the diameter ranges for the triglyceride-rich lipoprotein (TRL—formerly known as very-low-density lipoprotein or VLDL) particles, low-density lipoprotein (LDL) particles, and high-density lipoprotein (HDL) particles, as well as their subclasses, have been previously published [36]. Total cholesterol, HDL cholesterol, triglycerides, and apolipoprotein B (ApoB) were measured, and LDL cholesterol, non-HDL cholesterol, TRL cholesterol and TRL triglycerides were calculated using the Extended Lipid Panel Assay [36]. Details of the NMR quantification of the branched-chain amino acids (BCAA) and ketone bodies have been reported previously [36]. Assay development, analytical performance, and clinical validation of GlycA, lipoprotein insulin resistance index (LP-IR) (0–100; least to most insulin resistant), and diabetes risk index (DRI) (1–100; lowest to highest risk of developing type 2 diabetes) have also been reported [37,38]. Endpoint plasma samples were missing for two participants in each of the placebo and blueberry groups. Therefore, the final number of subjects for the analysis of the NMR-measured metabolites was 15 and 19 for the blueberry and placebo groups, respectively (Figure 1).

### 2.5. Statistical Analyses

For the fecal microbiome characterization, statistical tests were carried out using R version 4.2.0 [29]. The ‘stats’ R package was employed for Fisher’s exact tests, Wilcoxon rank-sum tests, and Wilcoxon signed-rank tests [29]. The ‘vegan’ R package was used to calculate the alpha and beta diversity indexes and permutational multivariate analysis of variance (PERMANOVA) [39]. The Shannon index (a measure of alpha diversity) was estimated for each sample using the genus and species level relative abundance profiles. The Bray–Curtis dissimilarities between the samples were calculated on both the genus and species taxonomic levels and visualized using principal coordinate analysis (PCoA). When applicable, *p*-values were Benjamini–Hochberg corrected for multiple testing. Differential abundance analysis of taxa between baseline and endpoint samples within the blueberry and placebo group were conducted using ANCOM-BC2 [40], separately. The ANCOM-BC linear models were constructed with age, gender, BMI category, and timepoint as the fixed effects and participant ID as the random effect. Taxa or pathways with prevalences (the proportion of samples in which the feature was present) <0.35 were excluded from the analysis. Benjamini–Hochberg correction was used to calculate adjusted *p*-values. Taxa with adjusted *p*-value < 0.05 were reported as statistically significantly enriched or depleted. Taxa with log2 fold change (L2FC) ≥ 1 were reported as enriched, and taxa with L2FC ≤ −1 were reported as depleted. The MetaCyc metabolic pathways of microbes in samples were predicted using sample 16S rRNA V3-V4 ASVs and their abundances using PICRUSt2 [41] with default options. Differential abundance analyses of the predicted metabolic pathways between baseline and endpoint samples within the blueberry and placebo group were conducted using ANCOM-BC2 [40], separately. Linear modeling and multiple hypothesis testing correction were performed the same as for differential abundance analyses of taxa. Pathways with an adjusted *p*-value < 0.05 were reported as statistically significantly enriched or depleted. Pathways with L2FC ≥ 0.5 were reported as enriched, and pathways with L2FC ≤ −0.5 were reported as depleted.

For the metabolite, lipid, and lipoprotein analyses, statistical analyses were conducted using SAS software version 9.4 (SAS Institute, Inc., Cary, NC, USA). Mean and standard deviation (SD) were calculated for analytes with a normal distribution, and median and interquartile range (IQR) were calculated for analytes with a non-normal distribution; change scores are the change in mean or median within-group. Within-group hypothesis tests were used to determine significant within-group differences in the change scores at 12 weeks; the paired T-test was used for analytes with a normal distribution, and the Wilcoxon signed-rank test was used for analytes with a non-normal distribution. Between-group hypothesis tests were used to determine significant between-group differences in the change scores at 12 weeks; the two-sample *t*-test was used for analytes with a normal distribution, and the Mann–Whitney U test was used for analytes with a non-normal distribution. *p*-values were considered statistically significant when *p* < 0.05 for both the within-group and between-group differences, and between-group significant findings with *p* < 0.0017 are bolded and italicized for emphasis.

## 3. Results

### 3.1. Clinical Characteristics of Study Participants

A summary of the demographic and clinical characteristics of study participants is found in Table 1. There were no statistically significant differences in age, gender, race, or BMI between the blueberry and placebo groups.

### 3.2. Fecal Microbiome Sequencing Results

**Figure 2:**
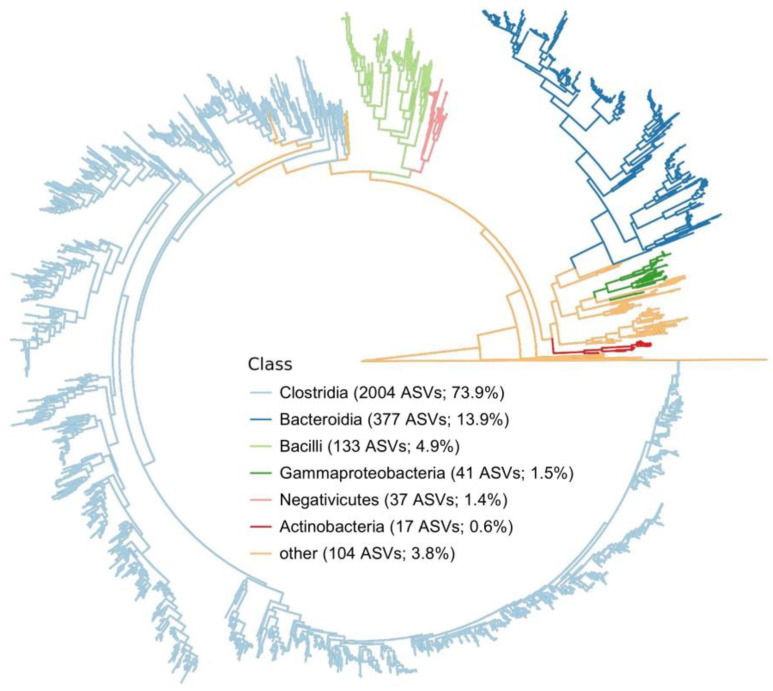
Maximum likelihood phylogeny of all amplicon sequence variants (ASVs), with branches colored by class. The number of ASVs and the proportion of ASVs in each class is noted.

**Figure 3:**
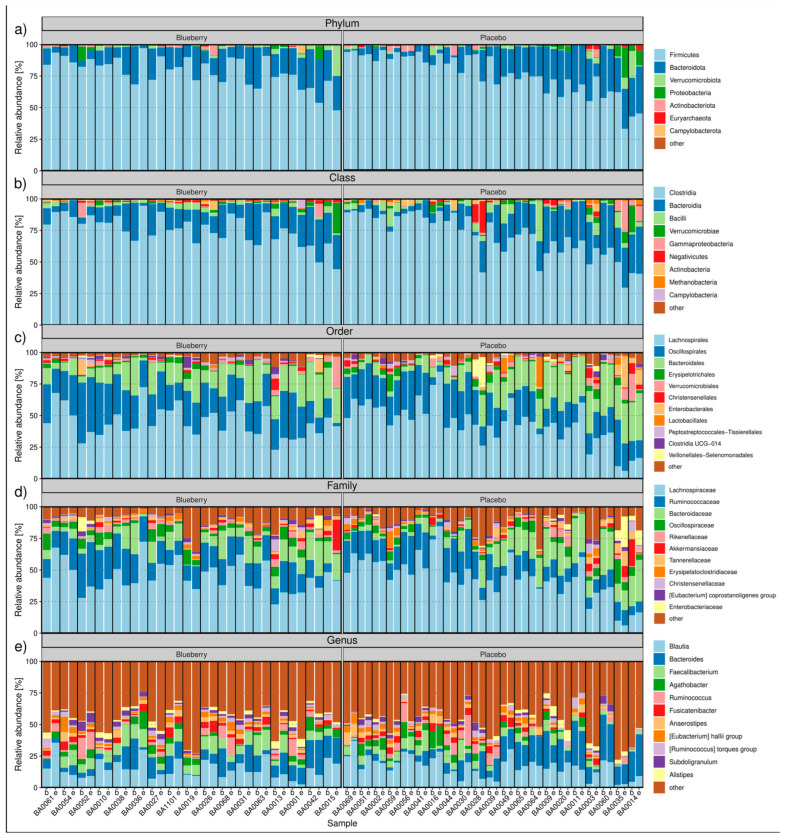
Stacked bar plot of relative abundances at (**a**) phylum, (**b**) class, (**c**) order, (**d**) family, and (**e**) genus taxonomic levels in blueberry (left of each plot) and placebo (right of each plot) samples. Samples were ordered by decreasing mean relative abundance of the most abundant taxa across baseline/endpoint, with participants separated by vertical lines. Each participant has baseline and endpoint samples grouped together from left to right. Taxa were sorted by mean relative abundance, and only those with at least 2.5% prevalence in at least one sample were kept; otherwise, they were aggregated in the “other” category. At most, the top ten taxa were shown in each plot, with all others aggregated in the “other” category.

Fecal swabs (n = 76) from 38 participants underwent 16S V3-V4 rRNA gene sequencing. All swabs were sequenced, with an average of ~91,400 genus-classified reads. Amplicon sequence variants (ASVs) (n = 2713; Table 2) were generated using DADA2, with most ASVs classified within the Clostridia class (2004 of 2713; 73.9%; Figure 2). As expected, Firmicutes and Bacteroidota were the dominant phyla across all 76 swabs analyzed (Figure 3a), Clostridia (2004/2713 ASVs) dominated at the class taxonomic level (Figure 3b), Lachnospirales dominated at the order taxonomic level (Figure 3c), and *Lachnospiraceae* dominated at the family taxonomic level (Figure 3d). Fecal microbiome profiles ranged from 10 to 60% of *Blautia* spp. and *Bacteroides* spp., with some minor differences between baseline and endpoint samples (Figure 3e).

### 3.3. Bacterial Composition and Diversity Analysis

**Figure 4:**
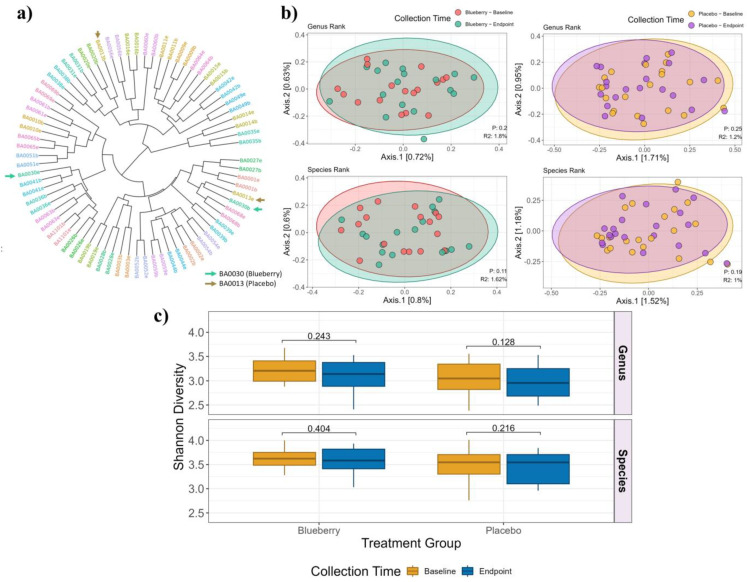
Composition and diversity analysis. (**a**) Samples clustered using pairwise Bray–Curtis dissimilarities of species-resolved microbiome profiles by participants. Microbes without species-level resolution were aggregated at the lowest taxonomic rank available. Tips were colored and labelled using the participant ID and then appended with either ‘e’ for endpoint or ‘b’ for baseline. Arrows represent samples from the same participant that were not clustered together. (**b**) Principal coordinate analysis of Bray–Curtis dissimilarity on genus and species rank colored by the blueberry (left) and placebo (right) treatment groups and time points. Filled ellipse represents 95% confidence ellipses. PERMANOVA with *p*-value and R-squared are labeled on each of the subfigures. (**c**) The Shannon diversity was measured at the genus (238 unique genera taxa) and species (380 unique species taxa) level for both blueberry and placebo groups at the baseline and endpoint. The boxplot shows the median, first, and third quartiles, and the whiskers indicate ±1.5 × interquartile range. The *p*-value is displayed on top of the paired comparison boxes.

**Figure 5:**
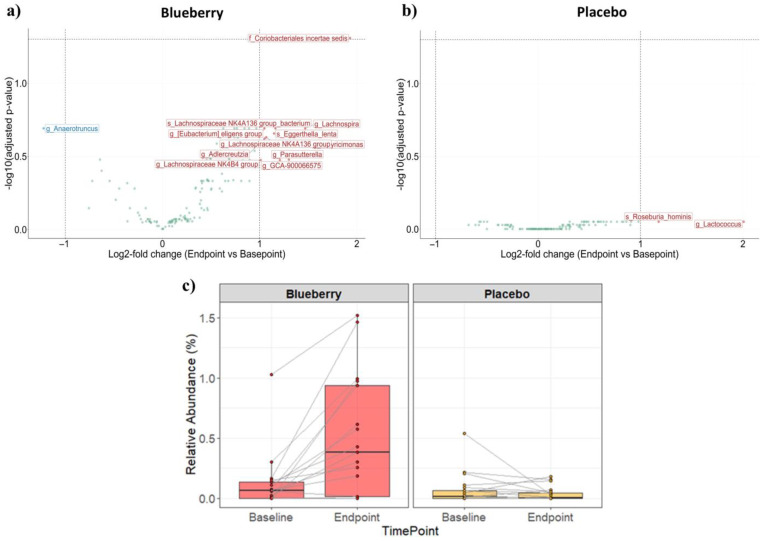
Differential abundance analysis of microbes. Volcano plots with log2 fold change (L2FC) (endpoint vs. baseline) on the *x*-axis and −log10-adjusted *p*-value (Benjamini–Hochberg correction) on the *y*-axis for two treatment groups; (**a**) blueberry group (left panel) and (**b**) placebo group (right panel). Data points represent taxa, which were aggregated using the lowest taxonomic rank classified for each amplicon sequence variants and are labeled with prefixes indicating the rank (‘s’ = species, ‘g’ = genus, ‘f’ = family). Colors indicate whether taxa are enriched (red, L2FC ≥ 1) or depleted (blue, L2FC ≤ −1) in endpoint samples relative to baseline samples. Light green taxa are those that did not have a large effect size (−1 < L2FC < 1). (**c**) Relative abundance of *Coriobacteriales incertae sedis* between baseline and endpoint samples for the blueberry group (left panel) and placebo group (right panel). Points represent individual samples. Lines connect samples from the same subject.

In order to measure the beta diversity, also referred to as the dissimilarity in bacterial composition between subjects or between groups, samples were clustered using pairwise Bray–Curtis dissimilarity indices. Baseline and endpoint samples clustered together in 36 of 38 participants, indicating that each study participant had a unique fecal microbiome signature at study start that was sustained after blueberry or placebo treatment (Figure 4a). However, no overall compositional differences in fecal bacterial genus or species were noted between the subjects in either the blueberry or placebo groups before and after treatment (Figure 4b). In addition, no significant change in the level of bacterial diversity was detected within either the blueberry or the placebo group between baseline and endpoint samples based on the Shannon diversity (Figure 4c). In other words, the fecal microbiome in the study subjects did not become more or less diverse with either blueberry or placebo treatment. Looking at individual bacterial taxa, however, *Coriobacteriales incertae sedis* was identified as the sole significantly enriched microbe in the blueberry group after treatment (adjusted *p*-value of 0.049) (Figure 5a), while no taxa were identified as significantly enriched or depleted in the placebo group (Figure 5b). Closer examination of the changes in the relative abundance of *Coriobacteriales incertae sedis* from baseline to endpoint within each subject in the blueberry group revealed it was enriched in 70.6% of participants (12 of 17), depleted in 11.8% of participants (2 of 17), and not detected in 17.6% of participants (3 of 17) (Figure 5c). No change in the relative abundance of *Coriobacteriales incertae sedis* was observed in the placebo group (Figure 5c). In the blueberry group, eleven taxa were enriched (including *Coriobacteriales incertae sedis*), and one taxon (*Anaerotruncus*) was depleted. In the placebo group, two taxa, *Roseburia hominis* and *Lactococcus*, were enriched, but the change did not reach statistical significance. None of the enriched or depleted taxa overlapped between the two treatment groups (Figure 5a,b).

### 3.4. Evaluation of Circulating Metabolic Biomarkers

To assess if a diet enhanced with blueberry or placebo powder was associated with changes in circulating biomarkers, including the gut microbiome-related metabolites TMAO, betaine, or choline, NMR was employed to provide data for a full lipoprotein profile, as well as targeted lipids and metabolites. In the fasting state, the placebo-treated group exhibited statistically significant reductions in total cholesterol (*p* = 0.019), LDL-C (*p* = 0.008), non-HDL-C (*p* = 0.022), total LDL-P (*p* = 0.008), large LDL-P (*p* = 0.015), and ApoB (*p* = 0.016) after 12 weeks compared to baseline. Participants whose diet was supplemented with blueberry powder exhibited significant reductions in total HDL-P (*p* = 0.021) and ApoA-I (*p* = 0.010) (Table 3). Similar results were obtained for samples collected in the post-prandial state (Table 4). Statistically significant differences between groups were noted in the fasting state for total LDL-P (*p* = 0.011), HDL size (*p* = 0.040), and ApoA-I (*p* = 0.030) (Table 3) and in the post-prandial state for LDL-C (*p* = 0.015), total LDL-P (*p* = 0.009), large LDL-P (*p* = 0.044), and ApoB (*p* = 0.033) (Table 4).

With respect to the gut microbiome-related metabolites TMAO, betaine, and choline, the placebo-treated group had a small but statistically significant increase in TMAO in the fasting state (*p* = 0.014) (Table 3), whereas there was a slight trend for a reduction in TMAO in the participants whose diet was supplemented with blueberry powder in both the fasting and post-prandial states (Table 3 and Table 4). There were no significant within-group differences in betaine or choline between baseline and 12 weeks in the fasting or post-prandial states, nor were there any statistically significant between-group differences in the three gut microbiome-related metabolites (Table 3 and Table 4). Similar conclusions could be drawn when considering all subjects with NMR data, with and without microbiome data, where the group sizes were larger—blueberry group (n = 28) versus placebo group (n = 27).

In contrast to the blueberry group, the placebo group had a significant reduction in total BCAAs in the fasting (*p* = 0.040) (Table 3) but not the post-prandial state (Table 4). However, no significant differences were noted in glucose, citrate, ketone bodies, or GlycA, a marker of systemic inflammation, between baseline and 12 weeks for either group. This was also true for the DRI and LP-IR scores (Table 3 and Table 4).

To assess if the change in the relative abundance of *Coriobacteriales incertae sedis* was associated with changes in the concentrations of the circulating biomarkers, including the gut microbiome-related metabolites (TMAO, betaine, and choline), Spearman correlations were calculated (Table 5). In the placebo-treated group, no significant correlations were noted between the relative abundance of *Coriobacteriales incertae sedis* and the circulating biomarkers, consistent with the lack of significant placebo group change in the fecal microbiome. In the blueberry-treated group, where there was a significant increase in the relative abundance of *Coriobacteriales incertae sedis*, no significant correlations between the change in the fecal microbiome and the circulating biomarkers were found in the fasting state. In the blueberry-treated group, a strong correlation between the increase in *Coriobacteriales incertae sedis* and a reduction in large LDL particles was observed in the post-prandial state. In the group that received the blueberry-enriched diet, increased *Coriobacteriales incertae sedis* was also associated with an increase in post-prandial isoleucine, DRI, and LP-IR scores. In the fasted state, a similar but not significant association was present for the change in fecal bacteria with isoleucine and the DRI scores. There were no significant associations between the change in *Coriobacteriales incertae sedis* and any of the other circulating biomarkers.

## 4. Discussion

The main conclusion of this study in older adults with overweight and obesity is that *Coriobacteriales incertae sedis* was enriched in the fecal microbiome of subjects who consumed a diet enriched with blueberry powder but not in those who consumed the placebo. In those with a blueberry-enriched diet, the increases in *Coriobacteriales incertae sedis*, a taxonomic group of bacteria that facilitates the metabolism of dietary polyphenols, likely reflect an adaptation to enable the digestion and absorption of blueberry polyphenols. Our finding of significantly enriched *Coriobacteriales incertae sedis* is consistent with previous rodent and human blueberry studies. Increased *Coriobacteriales incertae sedis* with blueberry consumption was observed in dietary studies in rodents [12,13,18]. In a rat model of postmenopausal estrogen deficiency, dose-dependent effects of blueberry consumption increased bacterial diversity in the fecal microbiome [18]. In addition, in this same rat model, four taxa, including two from the Coriobacterialis order, were significantly higher with blueberry treatment [18]. Also, in mice fed a diet enriched with Montgomery blueberries, bacterial diversity and *Coriobacteriales incertae* were increased [12]. Similarly, in high-fat-, high-sucrose diet-fed obese mice, addition of dietary wild blueberry polyphenolic extract led to increased *Coriobacteriales adlercreutzia equolifaciens* in fecal microbiota and improved glucose tolerance [13]. In humans, diets supplemented with blueberries, berry juice (including blueberry juice), or blueberry powder [24,25,26,27] resulted in changes in fecal microbiota, such as increased *Bifidobacteria* and other bacterial species thought to be associated with a healthy gut [24,25]. To our knowledge, ours is the only study in humans to show an increase in *Coriobacteriales incertae sedis* with increased blueberry consumption. Based on these and previous studies, the increase in *Coriobacteriales incertae sedis* may be a compensatory mechanism for increasing the digestion and absorption of greater amounts of dietary blueberry polyphenols. While the exact mechanisms are not well understood, this bidirectional relationship between dietary polyphenols and the fecal microbiome has been described previously [8,16,42,43,44,45,46,47].

While there was a high degree of diversity between individuals in terms of the abundance of various bacterial species, each subject had a unique fecal microbiome signature, and the 16S rRNA gene sequencing data show the fecal microbiome within individual subjects was consistent before and after treatment. This suggests that, in contrast to the blueberry supplementation studies in rodents, it may be challenging to observe significant changes in fecal microbiome diversity in groups with small numbers of human subjects because of the high interindividual diversity. This was evident in the current study, where we observed varied individual responses to blueberry powder (Figure 5c); some subjects exhibited no significant fecal microbiome changes, while others exhibited a significant increase in *Coriobacteriales incertae sedis* after consuming the blueberry-enhanced diet for 12 weeks. Aside from the one significantly enriched species of bacteria, the principal components of the microbiome species analysis showed no overall compositional differences between the baseline and endpoint fecal samples for either the blueberry or placebo groups. There was also no change in the fecal microbiome diversity (the microbiomes did not become more or less diverse), nor were any enriched or depleted pathways identified with blueberry compared to placebo powder supplementation.

No significant changes in the concentrations of the gut microbiome-related metabolites, TMAO, betaine, and choline, were observed after blueberry or placebo powder consumption. High-circulating TMAO concentrations are associated with increased cardiovascular and mortality risk, as well as renal dysfunction [20,21,22], and low levels of betaine are present in subjects with metabolic diseases, such as type 2 diabetes and nonalcoholic fatty liver disease (NAFLD, now referred to as metabolic dysfunction-associated steatotic liver disease or MASLD) [34,48]. Unlike in our study, in a previous study in mice, TMAO levels were reduced after 12 weeks on a diet enriched with blueberry, but not strawberry, powder [49]. To put our results into perspective, the subjects in this study had very low TMAO concentrations that were well within the normal range. Betaine and choline concentrations were also within their normal reference intervals. Therefore, while there was a small trend toward reductions in TMAO with blueberry consumption, we cannot conclude that these minor changes would lead to a reduction in cardiovascular risk.

Previous studies in individuals with and without chronic health conditions have shown improvements in cardiometabolic biomarkers following the consumption of blueberries [50]. Regarding HDL parameters, in a study by Curtis et al., non-statin users significantly increased HDL and ApoA-I when consuming 1 cup of blueberries per day [51]. In contrast to previous findings, the incorporation of blueberry powder into a dietary and exercise regimen led to a significant decrease in high-density lipoprotein (HDL) parameters, specifically total HDL particles (HDL-P) and ApoA-I, as observed in our research study. However, these changes were not specifically related to the increase in *Coriobacteriales incertae sedis*. The increase in *Coriobacteriales incertae sedis* in the blueberry group in the post-prandial state was correlated with a reduction in circulating large LDL particles. Because there was not a concomitant decrease in total LDL or small LDL, this change alone may not lead to a significant improvement in cardiometabolic risk. Based on the observed associations with increases in isoleucine, LP-IR, and DRI in the blueberry group in the post-prandial state, blueberry powder consumption may result in an increase in insulin resistance and diabetes risk over time. However, larger studies are needed to make this conclusion. There were no significant associations between the change in *Coriobacteriales incertae sedis* and any of the other circulating biomarkers. In addition, none of the changes in biomarker concentrations that were observed in the placebo group were correlated with a change in the fecal microbiome. Greater blueberry powder concentrations and/or a longer duration of blueberry treatment may be required to achieve health benefits in aging individuals with overweight or obesity and cardiometabolic risk factors than in healthy adults [50].

The strengths and limitations of this study are worth noting. The major limitation of this pilot study was the small group sizes for subjects with both fecal microbiome analysis and NMR-measured cardiometabolic biomarker analysis. However, similar conclusions could be drawn when considering all subjects with NMR data, with and without microbiome data, where the group sizes were larger. The strengths of this study include the novel measurement of human microbiome responses to blueberry consumption and the use of a placebo control group consuming an indistinguishable placebo powder and experiencing the same exercise regimen. In addition, the placebo and treatment groups were well matched in terms of age, gender, race, and BMI category. However, we did note differential responses in the placebo group. Placebo effects can be due to differences in trial motivation or lifestyle changes due to participants’ awareness of health interventions. Considering the favorable outcomes observed in the placebo group, it is plausible that individuals within this group exhibited behaviors influenced by these factors. While we do not currently have documentation of behaviors that could have influenced these variables in the placebo group, subsequent analyses will explore this question.

## 5. Conclusions

Regular consumption of blueberries may enhance the aging human gut’s capacity in individuals with overweight or obesity to effectively utilize dietary polyphenols. These findings suggest that, despite age-related effects, the gut maintains its ability to absorb and metabolize polyphenols with increased intake, thereby enabling older adults to derive maximum benefits from polyphenol consumption [52]. To further understand the implications of these findings for cardiometabolic risk factors in older adults with overweight and obesity, additional studies involving blueberries or blueberry powder, along with larger sample sizes, are warranted.

## Figures and Tables

**Table 1:** Demographics and clinical characteristics of study participants, with fecal microbiome results.

Characteristic	Overall (n = 38)	Blueberry Group (n = 17)	Placebo Group (n = 21)	*p*-Value
Age, years, mean (SD)	70 (65, 74)	71 (68, 77)	69 (63, 72)	0.2
Female, n (%)	19 (50)	7 (41)	12 (57)	0.5
White	33 (87)	16 (94)	17 (81)	0.4
BMI, 25–29.9 kg/m^2^, n (%)	20 (53)	10 (59)	10 (48)	0.5
BMI, ≥30 kg/m^2^, n (%)	18 (47)	7 (41)	11 (52)	0.5

n (%) for categorical variables; median (interquartile range) for continuous variables. Fisher’s exact test for categorical variable; Wilcoxon rank-sum test for continuous variables.

**Table 2:** Overview of fecal microbial features.

Microbial Feature/Taxonomic Rank	# Detected
Phylum	11
Class	17
Order	38
Family	65
Genus	195 *
Species	158 *
Amplicon sequence variants	2713

* Represents the unique taxa in a specific taxonomic rank. # = number

**Table 3:** Baseline values and change scores for NMR-measured lipid, lipoprotein, and metabolite levels measured at baseline and 12 weeks (Fasting) ^a^.

	Blueberry Group (n = 15)	*p*-Value ^b^	Placebo Group (n = 19)	*p*-Value ^b^	Between Group *p*-Value ^c^
Total Cholesterol (mg/dL)					
Baseline	191.1 (51.2)		188.1 (26.6)		
12 weeks	183.2 (42.5)		178.9 (28.5)		
Change at 12 weeks	−7.9 (20.2)	0.154	−9.2 (15.5)	0.019 *	0.833
TRL cholesterol (mg/dL)					
Baseline	26.0 (19.0, 41.0)		19.0 (12.0, 37.0)		
12 weeks	30.0 (18.0, 35.0)		20.0 (10.0, 36.0)		
Change at 12 weeks	−1.0 (−10.0, 2.0)	0.282	−3.0 (−6.0, 4.0)	0.516	0.768
LDL Cholesterol (mg/dL)					
Baseline	112.3 (46.4)		108.8 (19.1)		
12 weeks	110.5 (40.8)		99.6 (21.0)		
Change at 12 weeks	−1.8 (17.8)	0.701	−9.2 (13.4)	0.008 **	0.191
HDL Cholesterol (mg/dL)					
Baseline	56.1 (13.3)		61.3 (19.4)		
12 weeks	52.9 (11.5)		61.8 (21.0)		
Change at 12 weeks	−3.1 (4.3)	0.014 *	0.5 (6.8)	0.739	0.066
Non-HDL cholesterol (mg/dL)					
Baseline	135.0 (46.6)		126.8 (20.3)		
12 weeks	130.3 (40.6)		117.1 (23.3)		
Change at 12 weeks	−4.7 (19.3)	0.359	−9.7 (17.0)	0.022 *	0.436
Triglycerides (mg/dL)					
Baseline	105.0 (95.0, 134.0)		79.0 (59.0, 134.0)		
12 weeks	117.0 (79.0, 133.0)		92.0 (56.0, 145.0)		
Change at 12 weeks	−9.0 (−20.0, 8.0)	0.252	−4.0 (−23.0, 13.0)	0.401	0.677
TRL triglycerides (mg/dL)					
Baseline	75.0 (58.0, 111.0)		53.0 (39.0, 99.0)		
12 weeks	78.0 (50.0, 113.0)		60.0 (33.0, 109.0)		
Change at 12 weeks	−7.0 (−19.0, 2.0)	0.158	−1.0 (−20.0, 25.0)	0.615	0.579
Total TRL particles (nmol/L)					
Baseline	161.0 (65.7)		112.5 (58.7)		
12 weeks	147.6 (46.4)		111.1 (63.2)		
Change at 12 weeks	−13.5 (49.4)	0.308	−1.3 (47.6)	0.906	0.474
Very large TRL particles (nmol/L)					
Baseline	0.1 (0.0, 0.1)		0.1 (0.0, 0.2)		
12 weeks	0.1 (0.0, 0.2)		0.1 (0.0, 0.1)		
Change at 12 weeks	0.0 (−0.1, 0.1)	0.672	0.0 (−0.1, 0.0)	0.661	0.742
Large TRL particles (nmol/L)					
Baseline	1.5 (0.2, 5.1)		1.1 (0.3, 5.0)		
12 weeks	1.7 (0.5, 3.0)		1.1 (0.4, 6.0)		
Change at 12 weeks	0.0 (−1.3, 0.3)	0.278	0.0 (−0.9, 1.6)	0.678	0.454
Medium TRL particles (nmol/L)					
Baseline	16.0 (12.7, 37.8)		13.1 (8.5, 23.2)		
12 weeks	22.6 (12.0, 41.5)		14.7 (5.1, 27.7)		
Change at 12 weeks	0.7 (−2.7, 10.1)	0.590	0.6 (−6.9, 14.6)	0.709	0.945
Small TRL particles (nmol/L)					
Baseline	37.6 (7.8, 58.4)		20.3 (5.7, 48.6)		
12 weeks	35.2 (9.6, 42.2)		27.0 (4.2, 38.4)		
Change at 12 weeks	−11.5 (−32.8, 13.3)	0.358	−0.8 (−16.3, 27.5)	0.953	0.555
Very Small TRL particles (nmol/L)					
Baseline	61.1 (56.7, 97.7)		57.7 (21.6, 104.1)		
12 weeks	88.8 (70.4, 101.4)		47.2 (18.5, 110.1)		
Change at 12 weeks	0.2 (−44.8, 36.6)	0.966	−11.9 (−28.8, 33.4)	0.984	1.000
TRL size (nm)					
Baseline	43.4 (37.0, 53.7)		43.4 (41.4, 49.5)		
12 weeks	43.5 (39.0, 49.8)		47.2 (40.4, 49.3)		
Change at 12 weeks	0.5 (−2.9, 2.8)	1.000	−1.6 (−5.9, 6.2)	0.575	0.510
Total LDL-P (nmol/L)					
Baseline	1350.2 (522.5)		1390.9 (227.3)		
12 weeks	1389.7 (514.1)		1249.0 (251.3)		
Change at 12 weeks	39.5 (181.2)	0.412	−141.9 (208.1)	0.008 **	0.011
Large LDL-P (nmol/L)					
Baseline	216.0 (20.0, 345.0)		195.0 (60.0, 347.0)		
12 weeks	103.0 (26.0, 336.0)		122.0 (16.0, 318.0)		
Change at 12 weeks	−9.0 (−76.0, 18.0)	0.466	−60.0 (−140.0, 0.0)	0.015 *	0.155
Medium LDL-P (nmol/L)					
Baseline	402.0 (121.0, 825.0)		445.0 (277.0, 737.0)		
12 weeks	463.0 (320.0, 846.0)		390.0 (260.0, 656.0)		
Change at 12 weeks	91.0 (−57.0, 117.0)	0.389	21.0 (−150.0, 171.0)	0.891	0.755
Small LDL-P (nmol/L)					
Baseline	597.1 (353.6)		665.5 (424.0)		
12 weeks	643.8 (337.4)		604.3 (360.7)		
Change at 12 weeks	46.7 (296.5)	0.551	−61.2 (277.9)	0.350	0.287
LDL size (nm)					
Baseline	20.7 (0.7)		20.8 (0.6)		
12 weeks	20.6 (0.7)		20.7 (0.6)		
Change at 12 weeks	−0.1 (0.3)	0.384	−0.0 (0.4)	0.610	0.755
ApoB (mg/dL)					
Baseline	100.0 (26.8)		98.7 (12.4)		
12 weeks	99.6 (25.8)		93.2 (13.9)		
Change at 12 weeks	−0.4 (11.0)	0.890	−5.5 (9.0)	0.016 *	0.156
Total HDL-P (μmol/L)					
Baseline	21.4 (3.6)		21.2 (3.3)		
12 weeks	20.3 (2.9)		20.9 (3.3)		
Change at 12 weeks	−1.1 (1.7)	0.021 *	−0.3 (1.7)	0.490	0.155
Large HDL-P (μmol/L)					
Baseline	2.0 (1.7, 3.8)		2.5 (1.6, 3.5)		
12 weeks	1.9 (1.3, 3.2)		2.1 (1.6, 5.5)		
Change at 12 weeks	−0.1 (−0.9, 0.2)	0.273	0.3 (−0.3, 0.7)	0.079	0.066
Medium HDL-P (μmol/L)					
Baseline	2.6 (1.2, 3.8)		2.6 (1.3, 4.5)		
12 weeks	2.2 (1.2, 3.8)		2.2 (1.1, 4.2)		
Change at 12 weeks	0.3 (−1.0, 0.8)	0.773	0.0 (−0.9, 0.4)	0.755	0.466
Small HDL-P (μmol/L)					
Baseline	16.2 (2.9)		15.2 (3.9)		
12 weeks	15.2 (2.6)		14.7 (3.9)		
Change at 12 weeks	−1.0 (2.3)	0.111	−0.5 (2.2)	0.339	0.514
HDL Size (nm)					
Baseline	9.0 (0.3)		9.1 (0.5)		
12 weeks	9.0 (0.3)		9.2 (0.6)		
Change at 12 weeks	−0.0 (0.1)	0.313	0.1 (0.2)	0.074	0.040
ApoA-I (mg/dL)					
Baseline	132.5 (24.0)		136.5 (30.0)		
12 weeks	124.1 (20.3)		137.8 (31.4)		
Change at 12 weeks	−8.5 (11.0)	0.010 *	1.3 (13.9)	0.697	0.030
Total BCAA (μmol/L)					
Baseline	379.1 (74.3)		377.8 (76.1)		
12 weeks	368.5 (53.4)		355.4 (55.4)		
Change at 12 weeks	−10.6 (46.0)	0.387	−22.4 (44.1)	0.040 *	0.457
Valine (μmol/L)					
Baseline	218.5 (42.2)		219.6 (40.5)		
12 weeks	209.1 (30.1)		206.5 (26.4)		
Change at 12 weeks	−9.4 (34.6)	0.310	−13.2 (30.5)	0.076	0.743
Leucine (μmol/L)					
Baseline	107.7 (21.6)		101.5 (26.4)		
12 weeks	101.8 (20.5)		96.9 (27.7)		
Change at 12 weeks	−5.9 (14.9)	0.150	−4.5 (15.7)	0.225	0.801
Isoleucine (μmol/L)					
Baseline	53.0 (15.8)		56.7 (15.6)		
12 weeks	57.8 (11.8)		52.0 (13.7)		
Change at 12 weeks	4.8 (10.2)	0.089	−4.7 (14.1)	0.166	0.030
Alanine (μmol/L)					
Baseline	332.4 (76.8)		326.8 (82.6)		
12 weeks	348.2 (74.1)		348.9 (77.4)		
Change at 12 weeks	15.8 (66.8)	0.375	22.1 (65.6)	0.159	0.785
GlycA (μmol/L)					
Baseline	373.7 (54.8)		371.2 (58.0)		
12 weeks	382.0 (55.2)		358.8 (55.4)		
Change at 12 weeks	8.3 (42.3)	0.462	−12.3 (44.4)	0.242	0.178
Glucose (mg/dL)					
Baseline	113.0 (101.0, 115.0)		103.0 (90.0, 107.0)		
12 weeks	108.0 (105.0, 114.0)		96.0 (88.0, 118.0)		
Change at 12 weeks	0.0 (−6.0, 8.0)	0.796	−2.0 (−7.0, 6.0)	0.488	0.476
Citrate (mg/dL)					
Baseline	2.0 (2.0, 2.0)		2.0 (2.0, 3.0)		
12 weeks	2.0 (2.0, 3.0)		2.0 (2.0, 3.0)		
Change at 12 weeks	0.0 (0.0, 0.0)	1.000	0.0 (0.0, 1.0)	0.590	0.753
Total Ketone Bodies (μmol/L)					
Baseline	167.0 (130.0, 238.0)		210.0 (158.0, 304.0)		
12 weeks	164.0 (121.0, 279.0)		187.0 (137.0, 243.0)		
Change at 12 weeks	−7.0 (−55.0, 68.0)	0.989	−12.0 (−127.0, 10.0)	0.169	0.499
β-hydroxybutyrate (μmol/L)					
Baseline	98.0 (76.0, 135.0)		120.0 (89.0, 172.0)		
12 weeks	77.0 (63.0, 163.0)		112.0 (81.0, 144.0)		
Change at 12 weeks	−8.0 (−34.0, 31.0)	0.762	−1.0 (−61.0, 24.0)	0.615	0.890
Acetoacetate (μmol/L)					
Baseline	48.0 (37.0, 90.0)		57.0 (43.0, 96.0)		
12 weeks	56.0 (40.0, 98.0)		47.0 (40.0, 57.0)		
Change at 12 weeks	3.0 (−28.0, 26.0)	0.902	−5.0 (−41.0, 10.0)	0.206	0.205
Acetone (μmol/L)					
Baseline	17.0 (10.0, 38.0)		25.0 (14.0, 36.0)		
12 weeks	22.0 (18.0, 29.0)		21.0 (14.0, 25.0)		
Change at 12 weeks	1.0 (−12.0, 15.0)	0.729	−4.0 (−15.0, 6.0)	0.170	0.181
DRI (1–100)					
Baseline	33.5 (18.7)		31.6 (20.4)		
12 weeks	32.3 (16.2)		28.2 (19.2)		
Change at 12 weeks	−1.2 (9.9)	0.646	−3.5 (11.8)	0.217	0.546
LP-IR (score 0–100)					
Baseline	46.3 (15.8)		42.1 (19.6)		
12 weeks	45.9 (15.7)		43.2 (20.6)		
Change at 12 weeks	−0.3 (7.6)	0.867	1.1 (11.5)	0.694	0.675
TMAO (μM)					
Baseline	1.9 (0.1, 3.6)		0.5 (0.0, 1.9)		
12 weeks	1.0 (0.1, 4.3)		1.9 (0.7, 3.4)		
Change at 12 weeks	0.1 (−2.4, 3.4)	1.000	0.6 (0.0, 2.5)	0.014 *	0.314
Betaine (μM)					
Baseline	41.3 (37.2, 52.6)		40.1 (33.3, 47.6)		
12 weeks	40.0 (36.0, 56.3)		40.9 (35.1, 52.7)		
Change at 12 weeks	−1.3 (−4.7, 4.6)	0.689	2.5 (−4.6, 5.3)	0.449	0.366
Choline (μM)					
Baseline	9.9 (2.1)		8.7 (3.5)		
12 weeks	8.8 (3.0)		9.1 (3.2)		
Change at 12 weeks	−1.2 (3.9)	0.273	0.4 (3.4)	0.600	0.229

Abbreviations: ApoA-I, apolipoprotein A-I; ApoB, apolipoprotein B; BCAA, branched-chain amino acids; DRI, diabetes risk index; HDL, high-density lipoproteins; LDL, low-density lipoproteins; LP-IR, lipoprotein insulin resistance index; TMAO, trimethylamine-N-oxide; TRL, triglyceride-rich lipoproteins. ^a^ Values are the observed mean and standard deviation (SD) for analytes with a normal distribution or median and interquartile range (IQR) for analytes with a non-normal distribution; change scores are the change in mean or median within-group. ^b^ Within-group *p*-value tests for a significant within-group difference in the change score at 12 weeks and is calculated with the paired *t*-test for analytes with a normal distribution and the Wilcoxon signed-rank test for analytes with a non-normal distribution. ^c^ Between-group *p*-value tests for a significant between-group difference in the change score at 12 weeks and is calculated with the two-sample *t*-test for analytes with a normal distribution and the Mann–Whitney U test for analytes with a non-normal distribution. *, ** indicate within-group differences at the *p* < 0.05, 0.01 values, respectively. Between-group significant findings *p* < 0.0017.

**Table 4:** Baseline values and change scores for NMR-measured lipid, lipoprotein, and metabolite levels measured at baseline and 12 weeks (post-prandial) ^a^.

	Blueberry Group (n = 15)	*p*-Value ^b^	Placebo Group (n = 19)	*p*-Value ^b^	Between Group *p*-Value ^c^
Total Cholesterol (mg/dL)					
Baseline	195.6 (49.9)		198.9 (27.5)		
12 weeks	192.9 (46.9)		186.5 (29.5)		
Change at 12 weeks	−2.7 (20.1)	0.607	−12.5 (17.1)	0.005 **	0.146
TRL cholesterol (mg/dL)					
Baseline	34.0 (25.0, 42.0)		24.0 (16.0, 41.0)		
12 weeks	33.0 (24.0, 40.0)		21.0 (14.0, 38.0)		
Change at 12 weeks	−1.0 (−5.0, 3.0)	0.608	−3.0 (−5.0, 5.0)	0.459	0.689
LDL Cholesterol (mg/dL)					
Baseline	110.6 (45.1)		113.4 (21.3)		
12 weeks	112.1 (43.2)		100.5 (18.2)		
Change at 12 weeks	1.5 (17.0)	0.743	−12.9 (14.7)	0.001 **	0.015
HDL Cholesterol (mg/dL)					
Baseline	56.0 (13.3)		63.1 (18.7)		
12 weeks	53.6 (12.5)		63.4 (21.7)		
Change at 12 weeks	−2.4 (5.0)	0.085	0.3 (7.8)	0.862	0.228
Non-HDL cholesterol (mg/dL)					
Baseline	139.6 (46.1)		135.8 (22.1)		
12 weeks	139.3 (44.8)		123.1 (23.7)		
Change at 12 weeks	−0.3 (19.2)	0.947	−12.8 (17.9)	0.006 **	0.063
Triglycerides (mg/dL)					
Baseline	150.0 (132.0, 185.0)		111.0 (84.0, 159.0)		
12 weeks	141.0 (128.0, 191.0)		118.0 (73.0, 200.0)		
Change at 12 weeks	−6.0 (−22.0, 14.0)	0.836	−11.0 (−37.0, 30.0)	0.818	0.808
TRL triglycerides (mg/dL)					
Baseline	127.0 (110.0, 161.0)		91.0 (63.0, 123.0)		
12 weeks	123.0 (106.0, 158.0)		84.0 (53.0, 165.0)		
Change at 12 weeks	0.0 (−14.0, 14.0)	0.796	−4.0 (−40.0, 31.0)	0.775	0.903
Total TRL particles (nmol/L)					
Baseline	162.5 (55.9)		123.6 (61.2)		
12 weeks	151.8 (51.1)		110.7 (55.4)		
Change at 12 weeks	−10.7 (45.1)	0.375	−12.9 (48.7)	0.263	0.891
Very large TRL particles (nmol/L)					
Baseline	0.3 (0.2, 0.4)		0.2 (0.1, 0.4)		
12 weeks	0.3 (0.2, 0.7)		0.2 (0.1, 0.4)		
Change at 12 weeks	0.0 (−0.1, 0.2)	0.720	0.0 (−0.2, 0.1)	0.426	0.328
Large TRL particles (nmol/L)					
Baseline	3.1 (0.4, 7.4)		0.6 (0.0, 6.0)		
12 weeks	2.6 (0.1, 7.9)		1.1 (0.0, 11.7)		
Change at 12 weeks	0.0 (−2.5, 1.1)	0.850	0.0 (−0.4, 1.9)	0.252	0.413
Medium TRL particles (nmol/L)					
Baseline	30.4 (19.4, 39.2)		22.1 (13.8, 31.6)		
12 weeks	33.6 (8.2, 40.1)		22.5 (8.3, 38.1)		
Change at 12 weeks	−4.7 (−15.0, 10.3)	0.366	2.7 (−10.8, 6.9)	0.945	0.510
Small TRL particles (nmol/L)					
Baseline	40.8 (23.2, 72.7)		33.8 (18.2, 58.8)		
12 weeks	45.3 (20.1, 76.8)		29.5 (14.8, 41.0)		
Change at 12 weeks	−4.4 (−17.7, 20.1)	0.639	−5.9 (−29.9, 8.6)	0.210	0.781
Very Small TRL particles (nmol/L)					
Baseline	73.8 (36.1, 103.3)		48.1 (24.4, 99.6)		
12 weeks	54.5 (49.4, 77.7)		43.9 (12.3, 97.7)		
Change at 12 weeks	11.5 (−31.1, 31.0)	0.847	−12.1 (−23.5, 24.5)	0.609	0.405
TRL size (nm)					
Baseline	44.9 (38.7, 51.6)		41.2 (36.2, 51.1)		
12 weeks	42.6 (38.8, 55.4)		41.5 (37.7, 51.3)		
Change at 12 weeks	0.9 (−4.1, 4.1)	0.902	1.0 (−4.0, 3.6)	0.945	1.000
Total LDL-P (nmol/L)					
Baseline	1349.0 (498.4)		1405.1 (238.7)		
12 weeks	1409.9 (539.4)		1271.5 (230.5)		
Change at 12 weeks	60.9 (204.7)	0.268	−133.5 (194.3)	0.008 **	0.009
Large LDL-P (nmol/L)					
Baseline	218.0 (9.0, 357.0)		249.0 (82.0, 450.0)		
12 weeks	110.0 (29.0, 372.0)		153.0 (53.0, 329.0)		
Change at 12 weeks	0.0 (−45.0, 72.0)	0.843	−117.0 (−151.0, 12.0)	0.002 **	0.044
Medium LDL-P (nmol/L)					
Baseline	343.0 (194.0, 721.0)		407.0 (249.0, 626.0)		
12 weeks	519.0 (139.0, 794.0)		385.0 (201.0, 680.0)		
Change at 12 weeks	−37.0 (−146.0, 218.0)	0.454	−23.0 (−105.0, 212.0)	0.679	0.986
Small LDL-P (nmol/L)					
Baseline	669.5 (313.5)		681.6 (355.7)		
12 weeks	691.8 (328.7)		611.1 (346.4)		
Change at 12 weeks	22.3 (180.7)	0.639	−70.6 (269.3)	0.268	0.239
LDL size (nm)					
Baseline	20.6 (0.7)		20.8 (0.6)		
12 weeks	20.6 (0.5)		20.7 (0.6)		
Change at 12 weeks	−0.0 (0.4)	0.951	−0.1 (0.3)	0.463	0.720
ApoB (mg/dL)					
Baseline	104.2 (26.4)		105.3 (13.7)		
12 weeks	105.3 (27.8)		97.9 (14.8)		
Change at 12 weeks	1.1 (11.4)	0.705	−7.4 (10.5)	0.007 **	0.033
Total HDL-P (μmol/L)					
Baseline	22.1 (3.5)		22.6 (3.1)		
12 weeks	21.4 (3.1)		22.1 (3.6)		
Change at 12 weeks	−0.7 (1.8)	0.147	−0.4 (2.0)	0.354	0.663
Large HDL-P (μmol/L)					
Baseline	2.3 (1.4, 4.0)		2.7 (1.5, 3.8)		
12 weeks	2.1 (1.3, 3.3)		2.2 (1.6, 5.5)		
Change at 12 weeks	−0.2 (−0.6, 0.3)	0.382	0.2 (−0.3, 1.0)	0.200	0.123
Medium HDL-P (μmol/L)					
Baseline	3.4 (1.7, 4.5)		3.3 (1.8, 5.1)		
12 weeks	3.1 (1.7, 4.8)		2.9 (1.9, 5.0)		
Change at 12 weeks	−0.4 (−1.1, 1.2)	0.730	−0.2 (−1.0, 0.4)	0.672	0.690
Small HDL-P (μmol/L)					
Baseline	16.3 (2.7)		15.8 (3.9)		
12 weeks	15.5 (2.0)		15.2 (4.1)		
Change at 12 weeks	−0.8 (2.0)	0.134	−0.6 (2.2)	0.273	0.736
HDL Size (nm)					
Baseline	9.0 (0.3)		9.2 (0.5)		
12 weeks	9.0 (0.3)		9.2 (0.6)		
Change at 12 weeks	−0.0 (0.2)	0.527	0.0 (0.2)	0.315	0.259
ApoA-I (mg/dL)					
Baseline	136.5 (22.7)		144.4 (28.7)		
12 weeks	131.1 (22.0)		144.4 (32.9)		
Change at 12 weeks	−5.3 (12.3)	0.114	−0.1 (15.2)	0.988	0.271
Total BCAA (μmol/L)					
Baseline	362.7 (79.3)		356.5 (68.9)		
12 weeks	349.3 (54.7)		333.4 (60.7)		
Change at 12 weeks	−13.3 (57.8)	0.387	−23.1 (52.5)	0.071	0.614
Valine (μmol/L)					
Baseline	213.6 (44.3)		207.2 (34.9)		
12 weeks	199.6 (28.2)		195.8 (24.9)		
Change at 12 weeks	−14.0 (31.8)	0.110	−11.4 (26.8)	0.081	0.799
Leucine (μmol/L)					
Baseline	95.7 (28.3)		94.1 (27.3)		
12 weeks	94.1 (21.2)		87.3 (30.1)		
Change at 12 weeks	−1.7 (26.5)	0.811	−6.8 (27.5)	0.293	0.582
Isoleucine (μmol/L)					
Baseline	53.2 (14.2)		55.5 (14.4)		
12 weeks	55.7 (15.7)		50.4 (15.4)		
Change at 12 weeks	2.5 (14.3)	0.515	−5.1 (13.6)	0.118	0.127
Alanine (μmol/L)					
Baseline	414.3 (86.6)		438.4 (90.4)		
12 weeks	425.1 (79.0)		456.8 (94.6)		
Change at 12 weeks	10.8 (53.7)	0.449	18.4 (72.7)	0.284	0.727
GlycA (μmol/L)					
Baseline	380.9 (62.7)		382.6 (68.6)		
12 weeks	389.9 (61.3)		364.8 (62.4)		
Change at 12 weeks	9.0 (45.0)	0.452	−17.8 (54.3)	0.171	0.126
Glucose (mg/dL)					
Baseline	100.0 (83.0, 113.0)		99.0 (78.0, 120.0)		
12 weeks	91.0 (81.0, 115.0)		98.0 (83.0, 109.0)		
Change at 12 weeks	0.0 (−9.0, 5.0)	0.822	−4.0 (−9.0, 9.0)	0.357	0.677
Citrate (mg/dL)					
Baseline	2.0 (2.0, 3.0)		2.0 (2.0, 3.0)		
12 weeks	2.0 (2.0, 3.0)		2.0 (2.0, 3.0)		
Change at 12 weeks	0.0 (0.0, 1.0)	0.313	0.0 (0.0, 0.0)	1.000	0.294
Total Ketone Bodies (μmol/L)					
Baseline	131.0 (107.0, 156.0)		135.0 (119.0, 145.0)		
12 weeks	128.0 (104.0, 145.0)		124.0 (105.0, 153.0)		
Change at 12 weeks	0.0 (−30.0, 6.0)	0.288	−6.0 (−44.0, 17.0)	0.390	0.808
β-hydroxybutyrate (μmol/L)					
Baseline	67.0 (61.0, 81.0)		80.0 (63.0, 91.0)		
12 weeks	73.0 (56.0, 83.0)		75.0 (54.0, 92.0)		
Change at 12 weeks	0.0 (−22.0, 15.0)	0.615	−2.0 (−17.0, 7.0)	0.401	0.822
Acetoacetate (μmol/L)					
Baseline	37.0 (32.0, 57.0)		39.0 (24.0, 56.0)		
12 weeks	41.0 (27.0, 46.0)		35.0 (22.0, 47.0)		
Change at 12 weeks	−2.0 (−16.0, 9.0)	0.479	−5.0 (−14.0, 9.0)	0.390	0.945
Acetone (μmol/L)					
Baseline	21.0 (12.0, 28.0)		17.0 (13.0, 21.0)		
12 weeks	17.0 (9.0, 27.0)		19.0 (13.0, 23.0)		
Change at 12 weeks	−3.0 (−17.0, 7.0)	0.349	1.0 (−7.0, 7.0)	0.962	0.435
DRI (1–100)					
Baseline	30.4 (17.3)		27.6 (20.4)		
12 weeks	30.4 (16.4)		23.2 (18.6)		
Change at 12 weeks	0.0 (13.1)	1.000	−4.5 (14.7)	0.201	0.356
LP-IR (score 0–100)					
Baseline	45.8 (17.3)		39.2 (25.1)		
12 weeks	45.1 (18.0)		38.7 (23.0)		
Change at 12 weeks	−0.7 (11.8)	0.813	−0.5 (11.3)	0.857	0.949
TMAO (μM)					
Baseline	2.0 (0.2, 5.2)		1.5 (0.4, 2.5)		
12 weeks	0.9 (0.4, 4.9)		1.5 (0.6, 3.7)		
Change at 12 weeks	0.3 (−4.7, 3.4)	0.639	0.0 (−1.4, 1.2)	0.984	0.755
Betaine (μM)					
Baseline	42.3 (38.5, 56.8)		39.3 (33.7, 49.1)		
12 weeks	44.9 (35.9, 58.4)		42.5 (39.6, 48.8)		
Change at 12 weeks	0.9 (−4.9, 3.3)	0.492	1.4 (−2.3, 6.4)	0.378	0.321
Choline (μM)					
Baseline	11.5 (3.1)		10.8 (3.1)		
12 weeks	10.1 (2.7)		10.2 (2.5)		
Change at 12 weeks	−1.4 (3.2)	0.103	−0.6 (2.8)	0.360	0.435

Abbreviations: ApoA-I, apolipoprotein A-I; ApoB, apolipoprotein B; BCAA, branched-chain amino acids; DRI, diabetes risk index; HDL, high-density lipoproteins; LDL, low-density lipoproteins; LP-IR, lipoprotein insulin resistance index; TMAO, trimethylamine-N-oxide; TRL, triglyceride-rich lipoproteins. ^a^ Values are the observed mean and standard deviation (SD) for analytes with a normal distribution or median and interquartile range (IQR) for analytes with a non-normal distribution; change scores are the change in mean or median within-group. ^b^ Within-group *p*-value tests for a significant within-group difference in the change score at 12 weeks and is calculated with the paired *t*-test for analytes with a normal distribution and the Wilcoxon signed-rank test for analytes with a non-normal distribution. ^c^ Between-group *p*-value tests for a significant between-group difference in the change score at 12 weeks and is calculated with the two-sample *t*-test for analytes with a normal distribution and the Mann–Whitney U test for analytes with a non-normal distribution. ** indicate within-group differences at the *p* < 0.01 values, respectively. Between-group significant findings *p* < 0.0017.

**Table 5:** Correlations between the change in the relative abundance of *Coriobacteriales incertae sedis* and change in cardiometabolic risk factor concentration after 12 weeks on blueberry-enriched or placebo diets.

Analyte	Blueberry Group				Placebo Group			
	Fasting		Post-Prandial		Fasting		Post-Prandial	
	ρ	*p*-Value	ρ	*p*-Value	ρ	*p*-Value	ρ	*p*-Value
Large LDL particles	−0.404	0.171	−0.763	0.002	−0.208	0.440	−0.034	0.900
Isoleucine	0.522	0.067	0.617	0.025	0.415	0.110	−0.282	0.289
DRI	0.544	0.055	0.634	0.020	0.194	0.471	0.079	0.770
LP-IR	0.291	0.334	0.695	0.008	0.035	0.898	0.205	0.447

Only significant correlations are listed in this table. Abbreviations: DRI, diabetes risk index; LDL, low-density lipoproteins; LP-IR, lipoprotein insulin resistance index. ρ = Spearmen correlation coefficient.

## Data Availability

Data may be made available upon request.

## Abbreviations

ApoA-I, apolipoprotein A-I; ApoB, apolipoprotein B; ASV, amplicon sequence variant; BCAA, branched-chain amino acids; BMI, body mass index; CVD, cardiovascular disease; DRI, diabetes risk index; HDL, high-density lipoproteins; LDL, low-density lipoproteins; LP-IR, lipoprotein insulin resistance index; NMR, nuclear magnetic resonance spectroscopy; TMAO, trimethylamine-N-oxide; TRL, triglyceride-rich lipoproteins.
